# To Uncertainty and Beyond: Identifying the Capabilities Needed by Hospitals to Function in Dynamic Environments

**DOI:** 10.1177/10775587211057416

**Published:** 2021-11-21

**Authors:** Rachel Gifford, Bram Fleuren, Frank van de Baan, Dirk Ruwaard, Lieze Poesen, Fred Zijlstra, Daan Westra

**Affiliations:** 1 Maastricht University, The Netherlands

**Keywords:** hospitals, organizational change, COVID-19 pandemic, well-being

## Abstract

Hospitals operate in increasingly complex and dynamically uncertain environments. To understand how hospital organizations can cope with such profound uncertainty, this article presents a multiple case study of five hospitals during the COVID-19 crisis in a heavily hit region of the Netherlands. We find that hospitals make adaptations in five key categories, namely: reorganization, decision-making, human resources, material resources, and planning. These adaptations offer insights into the core capabilities needed by hospitals to cope with dynamic uncertainty. Our findings highlight the need for hospitals to become more flexible without sacrificing efficiency. Organizations can accomplish this by building in more sensing and seizing capabilities to be better prepared for and respond to environmental change. Furthermore, transforming capabilities allow organizations to be more resilient and responsive in the face of ongoing uncertainty. We make recommendations on how hospitals can build these capabilities and address the core challenges they face in this pursuit.

## Introduction

Health care organizations face constant, rapid, and complex change. Most recently, the COVID-19 pandemic has upended normal life in such a way that the environment has become “dynamically uncertain” (Christianson & Barton, 2021) or Volatile, Uncertain, Complex, and Ambiguous (VUCA) (Nembhard et al., 2020). Consequently, health care organizations must rapidly adapt and adopt learning mindsets and become more flexible to cope with these uncertainties (Nembhard et al., 2020; Teece et al., 2016). However, health care organizations, particularly hospitals, are typically heavily institutionalized (Reay & Hinings, 2009) with slow decision-making structures (Agwunobi & Osborne, 2016) and are typically slow to adopt innovation (Cahan et al., 2020). As the pandemic severely tests the resilience of health systems and presents hospitals with unprecedented and complex managerial challenges (Nembhard et al., 2020), it is essential to aid organizations in understanding how they can effectively adapt and handle complex and dynamic uncertainties.

The novelty of the severe acute respiratory syndrome coranavirus 2 (SARS-CoV-2) disease and the vastness of the pandemic confront organizations with what Teece et al. (2016) describe as “unknown unknowns” (i.e., deep uncertainties), with hospitals playing a leading role in handling them during the pandemic (Martin et al., 2021). As the situation unfolds, health care organizations also come to face financial challenges (Martin et al., 2021), absenteeism and burnout of staff (Gold, 2020). Paired with uncertainty about relief measures (e.g., vaccinations), sudden increases in the local demand for COVID-care and how the pandemic will develop (e.g., new variants emerging), these challenges create profound dynamic uncertainty. As a result, organizations must continuously find ways to harness their internal resources to remain responsive to environmental changes (Teece et al., 2016). Yet, until now, most theorizing has focused on efficiency over flexibility (Al-Amin et al., 2016). Our study attempts to add to this literature by theorizing how organizations can become more flexible in the context of efficiency.

To explore how hospitals can best handle dynamic uncertainty, this article explores how hospitals respond to dynamic uncertainty as experienced during the COVID-19 crisis. To this aim, we conducted an exploratory, multiple case study in five hospitals in one of the most heavily hit COVID-19 regions in the Netherlands to assess what capabilities are needed to help hospitals to confront and recover from crises, now and in the future. Specifically, we apply a dynamic capabilities perspective (Teece et al., 1997) to investigate how hospitals can successfully cope with emergent and dynamic uncertainty while still sustaining their internal resources. By analyzing internal documents and conducting in-depth interviews, we were able to identify what capabilities hospitals need to adapt to uncertain environments. Our study contributes to the literature and practice in several ways. First, it is a first step in building a much-needed knowledge base regarding adaptations hospitals make, the challenges they confront in making them rapidly, and the capabilities they need to adapt to uncertainty in the future. Second, this knowledge base lays a foundation for a more theoretical understanding of dynamic capabilities and adaptations hospitals make in deeply uncertain environments that can be applied to comparably challenging situations in the future.

### New Contribution

With the onset of COVID-19, scholars have highlighted the need to revisit current organizational theories and revise them in light of the pandemic specific challenges (Greve, 2020). More specifically, we require a better understanding of how to prepare health care organizations for situations of high uncertainty (Hick & Biddinger, 2020; Nembhard et al., 2020). Our study takes up this call by adopting a dynamic capabilities (Teece et al., 1997) perspective to empirically analyze hospitals’ adaptations during the COVID-19 pandemic to offer insights into how hospitals can cope with ongoing and future uncertainty. Despite its attention in other industries, the dynamic capabilities framework has seen limited application in the public sector, and specifically the health care context (Agwunobi & Osborne, 2016; Pablo et al., 2007; Teece et al., 2016). Through this work, we rethink the notions of sensing, seizing, and transforming in the context of the COVID-19 pandemic and offer suggestions as to how hospitals can build these capabilities within current system-level constraints. Our study thus offers unique insights for scholarship and policymakers alike and can help us to better equip health care organizations in the face of increased uncertainty (Geiger et al., 2019).

## Theory

Teece et al. (1997, p. 516) define dynamic capabilities as “the firm’s ability to integrate, build, and reconfigure internal and external competences to address rapidly changing environments in which there is deep uncertainty.” For organizations that face dynamically uncertain environments, such as the recent COVID-19 crisis, dynamic capabilities provide opportunities to cope with ongoing uncertainty and to become stronger for future uncertainty. Examples of essential dynamic capabilities include, sensing, seizing, and transforming (Teece et al., 1997, 2016). The core of the dynamic capabilities framework is the responsiveness of an organization to the shifting, external environment (Eisenhardt & Martin, 2000). As such, the framework is particularly suited to understand organizational adaptation in the face of crisis and to shed light on how organizations can prepare for situations of increasing uncertainty.

The dynamic capability framework posits that dynamic organizations have the capabilities to “sense” and “seize” opportunities and respond to threats and engage in continued transformation. Teece et al. (2016) assert that a dynamic organization can engage in certain activities to harness this capability. For example, organizations can engage in scenario planning to “imagine possible futures” (Schoemaker & Amit, 1997) and adjust organizational decision-making accordingly and in preparation. However, in situations of heightened uncertainty, such as the case may be with life-threatening events that are unpredictable and come on quickly, sensing may be more difficult. For example, in the face of the COVID-19 pandemic, health care systems were unable to grasp the reality of the situation fully until they were responding to it (see also Christianson & Barton, 2021). Despite having contingency plans in place, and even in countries that experienced the onset of the pandemic at later dates, health care organizations were unable to imagine (and thus be prepared for) the scale and length of the current crisis. We therefore suggest that due to short-term orientations (Agwunobi & Osborne, 2016) and the unprecedented nature of the COVID-19 crisis (Nembhard et al., 2020), hospitals were unable to “sense” what was to come, even when it was unfolding in other places (e.g., China and Italy).

Still, sensing is only the beginning. The ability of organizations and management to “get things done” is an essential component in facing uncertainty. Seizing involves the mobilization or reconfiguration of resources to meet emergent needs (Teece et al., 2016) and to position the organization in a favorable position to the external environment (Teece, 2007; Teece et al., 1997). As COVID-19 demonstrates, the flexibility of such organizations, for example to modify their internal structures and reconfigure their human and operational resources, determines their ability to adequately respond to the crisis and meet emergent demands. In the face of such deep uncertainty (Teece et al., 1997), dynamic organizations are thus also flexible organizations. Organizations can become more flexible by building slack into the organization (Teece et al., 2016). Slack refers to excess resources, for example in the form of redundant employees, excess capacity, or excess capital (Nohria & Gulati, 1996) and are crucial to deal with the challenges of the 21st century because they allow organizations to adapt, change, and protect critical processes from environmental turbulence (Lawson, 2001). However, hospitals are not known for being flexible, and economic reforms have spurred an increase in market mechanisms (Currie & Spyridonidis, 2016) and competition across Western systems in recent years prioritizing efficiency (Hick & Biddinger, 2020; Reay & Hinings, 2009) over flexibility. Subsequently, due to ongoing efficiency-oriented reforms, ongoing financial constraints and resource shortages, hospitals likely have faced significant barriers to seizing during the Covid-19 crisis, particularly in their reconfiguration of human resources.

In addition to sensing and seizing, it is important that organizations also engage in constant transformation (c.f. “renewal”) (Teece et al., 2016). Particularly when facing deep uncertainty, organizations need to exhibit a commitment to improvement and be able to quickly reflect and subsequently alter practices. Adapting a so-called “learning mind-set” (Nembhard et al., 2020) is essential for organizations to come up with and implement innovative solutions to evolving challenges, such as the COVID-19-pandemic. Learning allows organizations to “build an organizational understanding and interpretation of their environment and to begin to assess viable strategies” (Fiol & Lyles, 1985, p. 805). While learning can be challenging during times of crisis, reflection is essential during emergent events to enable organizations to continually update their responses as new information is received. For example, during COVID-19 hospitals had to act quickly with little information (Nembhard et al., 2020), and it was essential that hospitals engaged in continual learning and reflected upon early decision-making to correct errors and to better align their future responses with the constantly shifting environment (e.g., as more information about the disease pathology and spread became available).

Management plays a key role in fostering learning (Teece et al., 1997) and needs to ensure that organizations depart from old routines and modes of operating that are unsuccessful (Teece, 2007), and work to build, foster, and renew their existing capabilities (Ambrosini et al., 2009; Pablo et al., 2007). Fostering a learning mind-set also emphasizes creativity (Agwunobi & Osborne, 2016) and quick problem solving, both needed in times of uncertainty, and enables staff the input and discretion to come up with new [more effective] solutions to ongoing and emergent problems (Greve, 2003) such as those brought on by the COVID-19 crisis. As the duration and scope of the crisis also remained unclear (Remuzzi & Remuzzi, 2020), there was an impetus for learning quickly so that organizations could remain resilient in the face of future waves and developments. Therefore, we believe that the crisis will have spurred continual learning and forced leadership to “learn by doing” to remain responsive to emergent situation.

## Methods

To understand how hospitals can sustain themselves in situations of dynamic uncertainty, we conducted an exploratory, multiple case study of hospitals in one of the most heavily hit COVID-19 regions in the Netherlands. We collected internal documents and media reports, and conducted interviews in five hospitals beginning in March 2020 and continuing until January 2021. This study is part of the exploratory phase of a larger research project investigating hospitals’ responses to the COVID-19 crisis and their effects on employees’ sustainable employability, funded by the COVID-19 program of the Netherlands Organization for Health Research and Development. Ethical approval was received for this study.

### Setting

As of August 2021, the Netherlands reports 10.941 confirmed COVID-19 cases and 104.11 deaths per 100,000 inhabitants (World Health Organization [WHO], 2020). The country has experienced a sharp first wave of infections in spring of 2020, primarily in the Southern regions, and an ongoing second surge of infections as of October 2020 (WHO, 2020). We conducted a case study in five hospitals in one of the most severely hit regions of the Netherlands during the pandemic, particularly in the first wave in spring of 2020. The hospitals in our study range in size from one of the largest in the country to one of the smallest in the country, in terms of the number of available beds, patients treated, and amount of staff. All hospitals are private, nonprofit organizations that provide outpatient and inpatient care and most have a 24-hour emergency ward (Kroneman et al., 2016). One is an academic medical center and four are general hospitals, of which two are top clinical hospitals. All hospitals are members of the same Regional Acute Care Network, through which Dutch health care organizations coordinate crisis responses.

**Table 1.** Data Sources of the Study.

Interview data (from September 2020 to December 2020)							
Staff function	Case 1	Case 2	Case 3	Case 4	Case 5	Total	Total pages
Executive	2	1	1	1	1	6	57
Senior	5	4	4	1	1	14	212
Medical leadership	3	2	1			6	87
Clinical	2	2		2		6	102
Supporting	1	1	1			3	43
Total	13	10	7	4	2	36	487
Archival sources (from February 2020 to December 2020)							
	Case 1	Case 2	Case 3	Case 4	Case 5	Total (pages/minutes)	
Minutes^a^	152	110	117		98	477 (pages)	
Internal evaluations	42					42 (pages)	
Blogs					29	29 (pages)	
Videos				571		571 (minutes)	

a For confidentiality reasons, we received action and decision lists rather than full minutes.

### Data Collection

The primary data sources in each of our five cases include internal documents of hospitals and interviews. See Table 2 for an overview of our archival and interview data. In collaboration with a liaison officer at each participating hospital, we collected all documents that were relevant to help us understand how hospitals adapt to the COVID-19 pandemic. Ultimately, we received crisis meeting minutes (action and decision lists) from the first wave (February 2020–June 2020) and second wave of the pandemic (September 2020–January 2021) in all five hospitals. In some hospitals, we furthermore acquired strategic plans, internal evaluations, policy documents, and internal communication (in the form of blogs and video messages) related to the COVID-19 pandemic. In total, documents used for this study constituted over 548 pages of written material and over 500 minutes of video (see Table 2).

**Table 2.** Key Adaptations Hospitals Undertook in Responding to the COVID-19 Pandemic.

Category	Description	Type	Type	Type
Decision-making	Specific to how decision-making was organized, creation of decision-making bodies (i.e., CBT), and adaptations in communication and information sharing.	Governance and structure	Policy	
Reorganization	Regarding the scaling of care (up or down), structural [re]design and repurposing of wards and units.	Scaling capacity	Restructure	Innovate
Human resources	Issues related to staff redeployment, repurposing, task and role expansion or deduction, and recruitment and training.	Redeploy	Expand	Recruit and train
Material resources	Related to the purchasing of necessary materials, technologies, devices	PPE	Medical equipment	Technologies
Planning	Plans and strategies to prepare for future waves and crises.	Protocols	Forecasting	Learning

*Note.* CBT = central crisis team; PPE = personal protective equipment.

A round of preliminary interviews (10) were conducted in two of the large hospitals in our sample by graduate students (March 2020–April 2020) to capture organizational responses during the height of the first wave. These interviews helped to orient us to the field and informed our primary data collection. Between September 2020 and January 2021, we conducted primary interviews with targeted respondents in all five hospitals based on the initial findings from the preliminary interviews and documents. In some organizations, we restricted the number of interviews to reduce burden on staff during the second wave of the pandemic. In total, 36 primary interviews were conducted with the board, management, medical leaders, and medical staff (see Table 2). Interviews helped us to tease out details of the adaptations made, and build context around these adaptations. Questions focused on how organizational decisions were made, what alterations were made between waves, where organizational attention was focused (and how this shifted) and what adaptations were considered important when coping with uncertainty. As we aimed to understand how organizations coped with the dynamic uncertainty introduced by the COVID-19 pandemic and identify what capabilities they needed to cope effectively, we asked respondents to reflect upon the challenges they faced and lessons they learned, as well as to highlight successes and failures. Respondents also commented on what they felt their hospital needed to cope with future waves and uncertainty. Speaking with stakeholders at different levels in the organization provided us with a good overview of the various issues, from several viewpoints. As interviewing continued through the second wave, respondents could compare responses to the first and second waves, highlighting internal learning and adaptation.

Interviews were conducted in English or Dutch with two interviewers (one native English speaker, one native Dutch speaker) and were recorded with permission. Interviews took place digitally or in person where possible. Interview recordings were transcribed verbatim. For each interview, the lead interviewer created a written summary immediately after the interview concluded and both the lead and second interviewer took extensive notes during the interview. All respondents were offered the opportunity to member check their transcript.

### Analysis

Data were analyzed in line with a grounded theory approach (Strauss & Corbin, 1998), allowing the data to lead analyses. Our full data set included archival documents and communications, and interviews. Documents and interviews were uploaded into Atlas.ti and Microsoft Excel for organization and analysis. We catalogued hospitals’ adaptations based on the document, communications, and interview data (see online supplement 1 for the full catalogue). Initially, documents and internal communications provided a good baseline of organizational context and decision-making that allowed us to tailor our interview guide and to explore any questions raised. For instance, some documents indicated decisions around certain topics such as implementing policy decisions or scaling back beds, but in practice, action was not taken or there was a delay between discussion and implementation. This allowed us to use specific probes to capture the dynamics around decision-making and the implementation of ideas, and to uncover areas where challenges were present.

As we conducted interviews, we constantly iterated between insights from the documents and organizational insiders to create a full picture of organizational response. The catalogue began with an extensive list from the documents and was expanded and refined throughout the interview process. The two lead interviewers worked in tandem to identify a complete set of adaptations, organizational responses, and related issues that were raised in interviews. For these purposes, documents and interviews were reviewed and analyzed in depth. The two researchers engaged in ongoing discussion regarding the emergent list and insights, and cross checked each other’s work for validation and consolidation. In a second phase, adaptations were categorized into a smaller set, and we compiled associated lists of challenges and successes for each category. After we had initial categorizations, these were shared with the research team (seven researchers in total). From this discussion the categories were further refined and we went further with thematic analysis (Miles & Huberman, 1994). An early draft of the categories and lessons learned was sent to organizations for member checks and for another round of discussion in the research team. Based on all feedback, we consolidated categories into a catalogue that was shared with all organizations. This full catalogue has been made available open source (Gifford et al., 2021).

Through a final stage of thematic coding, we further analyzed this catalogue representing what organizations “did” to better understand what dynamic capabilities were needed by organizations in response to dynamic uncertainty. Here we begin assessing the capabilities held and needed by organizations to support adaptation. We also created an associated list of lessons learned (to indicate the presence of learning behaviors) and highlighted key issues for future consideration (to encourage learning) alongside the adaptation list. Examples of organizational learning could be identified via the comparative analysis between documents from the first wave of the pandemic (February–August 2020) and the second wave (September–January 2021). Interviewees also commented directly on the changes made as a result of lessons learned in the first wave. Subsequently, we developed the list of categories, descriptions and types as presented here (see Table 2 for an overview).

## Findings

In the following sections, we present the categories resulting from our final analysis and detail the key adaptations taken by organizations in the face of crisis (see Table 2). Each category highlights how organizations responded to a situation of dynamic uncertainty. Moreover, it offers information regarding where organizations had or have developed necessary capabilities (e.g., the ability to reconfigure resources) and insights into the dynamic capabilities that they might need, also for future crises (e.g., centralizing capacity planning, improving forecasting and decision-making). In each section, we highlight the lessons learned within our case hospitals to showcase the organizational learning during the pandemic. In our discussion, we integrate our findings and draw upon the lessons learned to offer a framework for action, detailing recommendations for organizations to build and harness necessary capabilities.

### Decision-Making

#### Governance and Structure

Part of the initial response to the COVID-19 crisis included switching the organizational governance model to activate a crisis structure. In the first wave, all hospitals created a crisis team structure that centralized decision-making. The composition of the crisis structure varied slightly across all hospitals but included a strategic central crisis team (CBT) and operational crisis team (OCT) in all cases. Members of the crisis structure included board members, departmental managers (e.g., HR, capacity planning) and medical leaders. The crisis team was dissolved in most hospitals following the end of the first peak of infections (between April to June 2020) and was subsequently incorporated into the normal hospital governance structure, working underneath or parallel to the board. In the second wave, some organizations expanded or altered the crisis structure to include formerly underrepresented groups such as nurses and medical specialists, and to give more responsibility to departmental level and line management. In association with this, many additional “COVID teams” were created from the beginning of the crisis, including COVID medical teams made up of physicians who focused on clinical protocols and medical decision-making.

#### Policy

An important element of decision-making was the creation or adaptation of existing policy to enable hospitals to respond efficiently to the emergent situation. The ability to make adaptations locally differed depending on the type of policy, for example if set by the national government (e.g., mandating that regular care remain at 80% during the second wave), professional organizations, regional bodies or individuals. Most adaptations in the first wave were taken [or subsequently translated] at a local level. In the studied region, one key adaptation decided regionally concerned the allowance of visitors in the hospital. Eventually, all hospitals restricted visitors. However, this had an effect on the other adaptations such as human resources. In the second wave, many policies became more centralized with national organizations, such as the creation of the national coordination center to coordinate patient transfers and IC capacity. Policy changes also concerned reconfiguring of human resources, such as getting permission to extend normal working hours (as outlined in the collective labor agreements; CAO) and frequency of shifts, allowing residents to work on the wards, hiring residents on temporary contracts, and extending clinical permissions such as ability to prescribe medication.

#### Lessons Learned

Given the emergent nature of the pandemic, hospitals had to make many rapid adjustments, and information was constantly incoming and being updated. Respondents stressed that it was important that leaders adopted a learning mind-set to respond effectively to the emergent situation and to be willing to “course correct” as needed.

I think it’s also a little bit of a mindset. You have to be creative and be prepared for the unprepared. I think it’s something thats not easy to run. We see also that you have it or you don’t have it and I think it costs a lot of time to find information. Normally when there was a fire there’s a fire and everybody sees the fire, [but Covid-19] this was not seen. . . you have to adapt very quickly to information and make a decision, but you also must be clear that you can be misinformed and that you have to turn something back and take another road [and] that’s not very easy for everyone. Crisis manager, C1

In the onset of crisis, hospitals felt it was easy to switch into crisis mode. The need for a crisis structure was apparent, and organizations perceived it as a key enabler of being able to take quick decisive action. However, in the second wave, organizations struggled with knowing when to implement the crisis structure, and changes led to confusion. Changes also led to tensions between quick decision-making and better involvement of all staff. As a result, adaptations were taken in the second wave to improve communication, for example, better feedback loops from the medical staff (e.g., regarding needs, updated clinical knowledge) were incorporated to capture important information and advice from medical staff.

### Reorganization

#### Scaling Capacity

All hospitals in our sample went through several similar phases of scaling their capacities. In the first wave, organizations had to scale up capacity rapidly for COVID-19 care, in particular by increasing ICU and Emergency care (A&E) capacity. To accomplish this, organizations all fully scaled down regular care between March and May 2020. From the end of April and May, organizations then began scaling back up regular care, maintaining COVID-19 care, and working to maintain a balance between the two throughout the second wave. In scaling down regular care organizations repurposed wards, redeployed staff, transitioned staff to work from home, and delayed patient care. Capacity was also increased, particularly in the ICU and acute wards, to allow for influx of patients and surgical (OR) capacity was restricted to allow human resources to match increased beds. In this category, capacities were quickly and widely reconfigured in response to the crisis. This signaled seizing capabilities but indicated a lack of overall sensing as adaptations often came as a result of immediate demand (i.e., when COVID-19 reached the Netherlands) rather than in preparation for (when cases were increasing in Italy).

#### Restructuring

Wards were restructured into COVID and non-COVID wards to keep disease spread down. All hospitals in our sample organized wards and flows into so-called “clean” and “dirty streams,” which helped to clarify the processes and protocols in place on wards. To make room for COVID care, certain wards were evacuated in some hospitals (e.g., psychiatric ward) and where wards were not in use, they were often repurposed. Some wards were turned into COVID wards or used to increase capacity for screening; others were repurposed for family members of patients, or as areas for staff to put on personal protective equipment (PPE). In addition, some outpatient clinics were reconfigured as digital wards for ongoing [virtual] regular care. In some hospitals, overflow units were built externally (outside hospital walls), for example to screen suspected patients or to create additional capacity.

#### Digitalization and Innovation

To allow regular care to continue as much as possible, hospitals engaged in digitalization and innovation. Here, we see the transition to E-health, video and phone consultations, and in some organizations the repurposing of wards or outpatient clinics to “digital” wards. Digitalization was also used for COVID patients to allow contact with family and medical staff, particularly in lieu of the no visitor policy implemented during the first wave. Innovations accompanied the scaling up of COVID care, for instance automating processes to reduce staff burden and improve speed (e.g., microbiology) and testing capacity. While such innovative solutions for care delivery have been possible, the crisis forced organizations to seize such opportunities, also in their regular services, more quickly.

##### Lessons Learned

After the first wave, hospitals recognized unanimously that scaling down regular care so significantly should be avoided in the future, indicating a previous failure to sense future challenges (and opportunities) due to a limited focus on present issues. Postponement of care led to additional burden for staff who had to work diligently and produce beyond normal limits to tackle waiting lists in periods where COVID-19 infections were low,

And looking back, but that’s always looking back. I think that it wasn’t necessary to cancel everything. The operations, yes. But for instance, all the X-rays, we stopped unless they were absolutely necessary, which meant that it was a very big backlog later. Physician, C2

Postponing care also prompted considerations of duty of care to patients. Professionals felt it was unethical to continue to delay care for those patients in need, sometimes multiple times throughout the year. Following the first wave, organizations worked to scale up regular care as much as possible within system limits, in some cases scaling beyond 100% of regular production. Hospitals and specialists felt this was imperative to deal with the waitlists and treat most needy patients. Financial implications were mostly indicated indirectly and differed by size of the organization.

### Human Resources

#### Redeployment

To match increased capacity for COVID, staff needed to be redeployed to other wards such as intensive care, emergency care, and to staff regular COVID-care wards. Staff unqualified to perform medical tasks were redeployed to supporting and coordinating roles, and administrative functions. Where staff were not able, or willing to be redeployed for COVID care, they worked at home or could support in additional ways (such as on the COVID support hotlines set up in some organizations). The redeployment of staff was a central concern, and challenge, for organizations throughout both waves. In the first wave, staff were often redeployed to new departments and teams on short notice, and were switched across roles and departments frequently. In the second wave, redeployment was more difficult, given the balance of regular care and resistance of staff to work on COVID wards.

In the first wave that was a lot of togetherness. Everyone helped each other and now, in the second wave I find it really different, in the sense that everyone goes for their own, their own [interests]. And on the one hand it is also understandable, because your own work also continues, but it is very unfortunate. Medical director, C3

#### Role Expansion

Throughout the organization, staff had to alter or expand their roles. This included management, clinical staff, and residents. Management were asked to step into different functions and take on tasks, for example in crisis management, communications, and crisis response. Clinical staff were asked to expand their patient ratios, responsibilities for other staff members (e.g., supervising less trained staff on clinical wards), extend their normal working contracts and hours, and general responsibilities for patient care such as prescribing medicines and in their clinical tasks. In addition, residents were given supervisory responsibilities of COVID wards and were in some cases hired by the hospital as employees to fulfill staff shortages and meet demand.

#### Recruitment

A central issue in the crisis response was recruiting enough staff to cover the increased demand of COVID care and other emergent demands as the crisis continued. External staff and volunteers were recruited, by putting out calls to recently retired staff or anyone with clinical registrations. Staff who had clinical backgrounds but were not currently working in clinical roles (e.g., working in administrative or managerial functions) were sourced internally. However, in some organizations, it proved difficult to place recruited staff and a clear strategy was not always in place. In the first wave, recruitment primarily related to covering the increased demand of COVID care, but in the second wave, regular care also restarted and had to be balanced, which exacerbated the burden on staff and the issues of staff shortages, requiring increased flexibility and good matching and recruitment strategies. The ability to redeploy staff remains an issue (particularly in the balancing of regular and COVID care) and shortages in key areas such as the ICU remain. Increased absenteeism due to quarantine necessity, infection, and other complaints, was also an issue which emerged into the second wave of the crisis and exacerbated existing constraints.

#### Training

To increase human resources, staff were trained for new functions and COVID care, such as performing intubations and working with ventilators and respiratory equipment. Training took place formally at in-house learning centers or academies, via daylong or multiple day training sessions, or via on-the-job training. In some organizations, buddy systems were created to pair experienced staff with newly trained staff. Following the first wave, training courses were also planned to upskill staff to support nursing functions and to increase flexibility for future crisis responses.

#### Lessons Learned

Staffing emerged as a central area of attention and importance throughout the crisis. Many organizations struggled in the beginning to redeploy staff in high-demand areas or to overcome shortages. Therefore, more attention was given to the competencies of human resources following the first wave.

I think we have to prepare better our human resources for these kind of situations . . . and also our purchasing departments, that are kind of lost departments that are not ready or prepared to do crisis management and to think out of the box. Manager, C1

In organizations that already had an overview of available staff and their clinical abilities, recruitment and redeployment was made much easier, demonstrating the importance of keeping central records of all staffs’ clinical backgrounds, certifications, and trainings. Organizations recognized that redeployment of staff in the first wave was often ad hoc, last minute, and that staff—especially nurses—were shuffled around frequently and without warning.

Looking back, we asked maybe too much from all sorts of regular staff. . . For instance, an operation nurse who usually gives instruments to the surgeon was now transferred to the intensive care unit and was doing other jobs and also saw people dying, which they usually do not do. Physician, C2

Organizations recognized the burden this put on staff, also due to the loss of support from a central team of peers. In the second wave, organizations strived to make more consistent redeployments and work on a voluntary basis. Organizations were still working to find ways to provide respite to staff by implementing better staffing protocols and training more staff to build slack into the organization. This remains an issue for further consideration.

### Material Resources

In response to COVID-19, hospitals needed to adapt the levels of supplies they had, how and where they sourced materials, and what resources they had in house. To match increased capacity and COVID demand, hospitals needed to increase the level of PPE, install plastic shields and protections, and source additional medical equipment and supplies (e.g., plastics for the lab). For example, some hospitals rented additional CT scanners for the COVID screening stations and overflow wards. Within this category, hospitals also had to make adaptations regarding who would get PPE, what PPE was used, and implement and follow [national] guidelines around PPE use. Shortages in PPE were felt most acutely in the onset of the first peak, where national coordination had not yet been well organized, demand was high, and the crisis trajectory was uncertain. Organizations faced dilemmas in making protocols about PPE use, for example between caring for patients and the safety of staff. While organizations made a commitment to protect staff; in practice, this was difficult due to considerations of patient care and safety. In some cases, staff had to work with PPE that was depreciating in quality, as some materials were being reused (washing and reuse of masks) and use lower-grade masks to save higher-grade masks for ICU and other COVID wards when shortages were high.

#### Lessons Learned

In the onset of the crisis, organizations were faced with material shortages and a global demand that created significant challenges to sourcing necessary materials. As a result, organizations sourced additional stock in the first wave beyond what they would normally keep. Going into the second wave with surplus was viewed as a protective factor that eased anxieties.

This situation in the second wave is much easier, much more organized, better for the patient, better for the personnel . . . [after the first wave] we knew a lot. We had our stock of materials we use for patients and personnel, we had enough, and in the first wave that wasn’t [the case]. It was every day a moment of counting of what we have and when is our stock [running out]. Manager, C2

Organizations sourced materials using both formal and informal networks, and in some cases, doctors even went directly to other professionals and community members to source masks (e.g., from dentists). The importance of informal networks emerged as a way to overcome nation-wide shortages at a local level. However, this introduces higher levels of variability into the system (e.g., at a regional level).

### Planning

Despite having undergone a virus preparedness training in October 2019, all hospitals considered themselves unprepared for such a large-scale and long-term event. High levels of uncertainty and a lack of protocols marked the situation, particularly in the first wave. As the disease itself was not well understood, professionals constantly had to search out and update clinical protocols. As cases in Wuhan and northern Italy rose in the beginning of the year (January–March, 2020), hospitals took their first preparations. Hospitals then continued to respond to the emergent crisis, and worked on creating better forecasting and capacity planning throughout the crisis. So called “code black” scenarios for what to do if patient demand exceeded resources were created in the first wave, for example, triaging care based on patient age. While these ultimately did not need to be implemented, these discussions prompted difficult and emotional ethical debates. Throughout the first wave and into the second, internal capacity management teams worked on forecasting to prepare the organization to scale capacity.

Maybe it’s not kind to say but we were not prepared for this. Because I think we never expected this could happen with this large impact on, especially at that moment, the ICU. Now we know that it’s also a bit because of all the [cut backs] in the personal staff and especially the nurses. . .And you saw it also in the availability of the ventilators, but also the protections, the masks, those kind of things. . . We are prepared for fire or maybe a bit of an ICT crisis, but not on this scale no. Hospital executive, C1

#### Lessons Learned

Based on their lack of sensing in the onset of the crisis, organizations recognized the need to create protocols and phased planning for crises. This includes crises beyond pandemics and other forms of disasters. In particular, hospitals recognized that despite prior crisis planning, they were unprepared for a crisis on a long-term scale (beyond a couple days), leaving them vulnerable in the wake of the current pandemic. Following the first wave, organizations began to put programs together and train support staff and nursing assistants to provide more flexibility in the system, focusing especially on building this capacity for human resources. Still, this remains an ongoing challenge. In the beginning of the crisis, informal networks between hospitals and health care providers played a big role in signaling what organizations should and needed to do, and prompted motivation for action internally. However, networks varied across organizations, meaning that different approaches and start dates are found across organizations.

## Discussion

Our study sought to understand hospitals’ adaptations in the face of the COVID-19 pandemic and the [dynamic] capabilities they require to engage in such adaptations. We find that hospitals engaged in five key categories of adaptations that provide us insights into the dynamic capabilities needed by organizations facing dynamic uncertainty: reorganization, decision-making, human resources, material resources, and planning. These adaptations correspond to the need of organizations to be flexible (e.g., reconfiguring staff and materials). As Teece and colleagues (2016) point out, “strong dynamic capabilities are necessary for fostering the organizational agility necessary to address deep uncertainty” (p. 13), including “sensing,” “seizing,” and “transforming.” In what follows, we discuss how organizations can develop these capabilities and become more flexible without sacrificing efficiency, a central paradox in the dynamic capabilities literature but essential in resource-constrained (Geiger et al., 2019) and unpredictable environments (Eisenhardt et al., 2010) like health care.

### Sensing

Respondents across all five hospitals indicated that their organizations were not sufficiently prepared for the COVID-19 crisis. This was partly attributed to the unprecedented nature of the crisis, and associated lack of information surrounding the disease pathology, treatment options, and trajectory. However, it also became clear that organizations had failed to recognize opportunities to develop their sensing capabilities, focusing instead on responding to more immediate environmental demands. This deficit was accentuated by the fact that all organizations had recently participated in a training in October 2019 to prepare for a potential biological threat. Respondents indicated that in spite of this training, and the imminent threat that viruses pose to humans, organizations were unprepared for any long-term and large-scale event such as the pandemic. Rather, they focused on crises such as accidents, ICT failures, terrorist attacks, and other short-term events. This aligns with the literature that asserts that organizations are often equipped with a short-term efficiency orientation (Zinn & Flood, 2009), which restricts their ability to sense opportunities (Agwunobi & Osborne, 2016).

#### Long-term Orientation

To be more resilient, a longer-term orientation at the strategic, tactical, and operational levels of decision-making is needed. While a short-term orientation emphasizes efficiency, a longer-term orientation allows organizations to build effective strategies (Wang & Bansal, 2012). In the face of crisis, organizations should make sure that a focus on sensing is built into their management functions. We therefore consider how organizations can be triggered to develop sensing capabilities, particularly when they tend to focus on the pursuit of short-term goals (Agwunobi & Osborne, 2016). Our findings show that in response to the uncertainty of COVID-19, there was a renewed focus on forecasting and prediction modeling (e.g., predicting likely cases based on testing data) within organizations. For example, in one organization, respondents indicated that the crisis helped organizations and medical professionals to recognize the crucial role that their operations and capacity management played. Capacity was used to being “owned” by clinicians who do not want to have this decided by another level of management, leading to trust issues between clinicians and management. However, during the crisis, by necessity, integral capacity management took a central role and worked on scenario modeling which helped to allocate beds to patients, and maintain continuity of care for patients needing operations and other services outside COVID care. This helped to build more goodwill and competence-based trust (Sako, 1992) from clinicians in this capacity, which can help foster further investment in this at the organizational level.

#### Integrating Functions

At the organizational level, capacity managers told us that they did not always receive instructions from the board about what to model and that the uncertainty of the situation left most people with “no idea” on what to do. This reveals that organizations need to better integrate internal competences (Teece et al., 1997). In particular, it teaches us that better integration between the operational core and technostructure (Mintzberg, 1979) is a way to trigger the incorporation of more sensing capabilities within organization. When these functions were better integrated, the day-today operations of clinicians was better connected to the bigger picture of the organization and environment as a whole. For example, physicians were considering how overall capacity matched with forecasted demand at the organizational level, rather than focusing on their own internal capacity as a separate function. Making this connection requires cultural changes (e.g., operating with short-term gains in mind, clinicians “owning” capacity management); however, with more trust and space in the system to develop this capacity, organizations can do more work to promote proactive strategies. Supporting structural elements are better forecasting capacities and information analysis (e.g., recruitment of capacity managers, data scientists) and employing top managers with a long-term orientation.

#### The Utility of Centralization

Rather than organizing sensing capabilities themselves, hospitals could benefit from centralized forms of information flow during crises, helping to increase strategic forecasting capacities (Baubion, 2013). Governmental or otherwise centralized databases may facilitate hospitals in sensing and responding appropriately during emergent crises, without the need for large investments at the organizational level. Such centralized forecasting is used in other types of crises, for example with the implementation of early warning systems, and can support the development of localized responses and adequately timed resource directions (Baubion, 2013). By formalizing the development (and later monitoring) of centralized information systems preventively, hospitals are less reliant on their informal networks and preparation can be optimized. However, the information flow between hospitals and such a centralized body must be bidirectional and will nonetheless require some investment on the part of individual organizations. This is necessary to maintain accurate information sharing that takes into account and allows for integration of local idiosyncrasies that may be response relevant. Furthermore, it is important to note that the development of such centralization mechanisms may face different barriers across health systems dependent on the role of the government in health care provision and regulation. For example, in systems where government plays a more centralized role (e.g., NHS systems), forecasting may be easier and more easily accepted by individual stakeholders.

### Seizing

To buffer against environmental threats (Zinn & Flood, 2009) and seize emergent opportunities (Teece et al., 2016), organizations can generate slack resources. The concept of slack implies that organizations accumulate excess resources that “allow the organization to forego short-term gains for long-term outcomes” (Zinn & Flood, 2009, p. 819). However, due to financial (restricted budgets), spatial constraints (hospital infrastructure), or system constraints (shortages of certain roles at a higher level), creating such excess might not always be possible in resource-constrained environments (Nohria & Gulati, 1996). The COVID-19 pandemic has revealed that hospitals have sacrificed much of this flexibility in the pursuit of efficiency, triggered by various efficiency-based reforms in the last years (Hick & Biddinger, 2020). They have “removed the air” from their systems (Zinn & Flood, 2009), making them unable to adapt to the uncertainty that contemporary challenges bring (Lawson, 2001). Nevertheless, our findings reveal two ways organizations can build slack and still maintain efficiency; through what we call “collective slack” and through increasing resource elasticity.

#### Collective Slack

Although theorization of slack resources typically occurs at the organizational level (Cheng & Kesner, 1997), our findings indicate that slack resources span organizational boundaries and therefore can be maintained at a collective (i.e., network or national) level. In fact, our results indicate that informal collective slack, formalized collective slack, and national collective slack constitute three distinct types of collective slack organizations can use. Two contextual examples illustrate this point. First, similar to many other countries (Cahan et al., 2020), the Netherlands faces shortages of clinical staff, in particular nurses (RTL Nieuws, 2020). From the onset of the pandemic, hospitals thus spent considerable energy on internal and external recruitment to build slack into their human resources. Clinical staff utilized informal networks to fill workplace gaps and meet increased demand and bed capacity, and organizations relied on informal networks of recently retired staff and local volunteers (informal collective slack). However, not all organizations had the same ability to recruit and use excess staff.

Having a pool of inactive, but qualified volunteers or redundant staff (Zinn & Flood, 2009) (formalized collective slack) that organizations can pull from would enable more resilience in the face of crises, and can help to alleviate the pressure of ongoing shortages outside of crises. An example of this is demonstrated in the growing collaboration between hospitals and the ministry of defense who take part in a program where core hospital staff are trained to provide care during difficult circumstances and become deployable during military missions (Baltesen, 2021). In exchange, professional medical soldiers work at hospitals when they are not on deployment. This is particularly useful for staffing intensive care nurses where shortages remain. As Cahan et al. (2020) suggest this may also be beneficial at the national level to alleviate persistent workforce gaps. Second, a similar “cascade” of slack sources was apparent in bed capacity, which has been a focal point throughout the pandemic (Cahan et al., 2020). In the absence of a coordinated system and in the face of bed shortages (particularly IC beds), medical staff initially used informal networks to arrange patient transfers (informal collective slack). Ultimately, a national coordination center (Landelijke Coordinatiecenrum Patienten Spreiding; LCPS) took control of capacity planning at a national level, even transferring patients to nearby countries that had excess bed capacity (national collective slack). A similar centralization mechanism was put in place for PPE resources at a national level, indicating the utility of building in collective slack at a higher level.

National governments can support organizations to become more dynamic by centralizing slack resources and working to build collective slack in both material and human resources. For example, national disaster preparedness organizations such as The Red Cross provide an example for an organizational mechanism that can recruit and train pools of volunteers. Governments may consider working with such organizations and professional associations to develop a national curriculum to train volunteers periodically for medically oriented emergencies and in basic functions that can help to supplement scarce resources, such as nursing assistants, crisis and first aid medicine, and so on. In addition, local governments or professional associations may consider registering a database of foreign residents that have been medically trained and certified in other countries to generate an additional volunteer pool.

#### Resource Elasticity

In the absence of slack resources and coupled with financial constraints the flexibility of human resources emerged as a central issue in our findings. In such circumstances, organizations can work to become more flexible by strategically augmenting their resources (Hick & Biddinger, 2020). Our findings reveal that organizations sought to make their human resource base more elastic in two ways; through training and upskilling existing staff and by delegating nonclinical tasks to volunteers and support staff. The training and upskilling of staff is needed, in particular, as a response to increased specialization of care. Specialization allows for more efficiency and an ability to handle increased workload (Argote & Greve, 2007) but restricts the flexibility of roles needed to respond to uncertainty and effectively redeploy resources. The upskilling and training of staff was thus an essential function for organizations, as existing staff did not have the flexibility (e.g., having the necessary qualifications to redeploy in high-demand areas) to cover the amount of demand in those areas. Training staff in more general and acute functions allowed them to offer support in the high-demand wards. In addition, shifting non-clinical tasks and non-complex tasks allowed existing staff to meet the increased demand for clinical work. Our findings suggest that organizations can become more flexible and cope with current and future uncertainty by having part of their staff engage in trainings and work rotations to maintain a broader base of general knowledge and skills so they can be redeployed more easily in crises. This can also help to counteract the difficulties faced by increasing specialization of staff such as poor collaboration, fragmentation, and organizational rigidity (Larson, 2017)

### Transforming

To renew and update their resource base, organizations need to learn. Learning is considered a precursor to building dynamic capabilities as well as a dynamic capability in itself (Ambrosini et al., 2009; Teece et al., 1997). Our findings illustrate learning behavior of organizations in each adaptation category. In all hospitals, evaluations were conducted following the first wave to get insights into how they could improve and alter their strategies and our findings show that all organizations respond differently to the second wave than they did to the first. For example, organizations did not scale down regular care as rigorously, adopted a different visitor policy, and altered training approaches for staff. Organizations also reorganized to allow space to continue to treat COVID patients alongside normal operations. Nevertheless, we find that organizations also struggled to retain the lessons learned and the positive gains of the first wave such as improved collaboration (between former siloes) and quick decision-making. Our results suggest that sustaining these changes requires a learning mindset throughout the organization that fosters higher-level learning and unlearning.

#### Higher-Level Learning

As recent scholarship has highlighted, when a system faces ongoing threats but returns to prior modes of functioning, the system becomes more vulnerable and thus less resilient to future challenges (Mithani, 2020). Our findings indicate that while practices shifted (indicating lower-level learning), overall norms and rules did not shift significantly enough to see long-term effects at the organizational level (Fiol & Lyes, 1985). To recover and become more resilient in the face of future threats (Greve, 2020), organizations need to engage in “higher-level” learning (Fiol & Lyes, 1985). This form of learning works to shift norms, helping organizations to develop new cognitive frameworks from which to make decisions and can help systems to unlearn (Starbuck, 1992) by discarding old frames. Such learning involves the alteration of existing mental models and associated practices and skillsets (Mithani, 2020).

#### Unlearning

Much of the change that organizations struggled to keep, such as improved collaboration between subunits, organizational cohesion, and investment in superordinate goals is likely to require cultural change. These changes, because they require the shifting of norms and historical “ways of being,” prove most difficult- particularly without the necessity and urgency (as we saw in the first wave of the crisis). Therefore, to become more resilient and develop better renewing capabilities, organizations need to “unlearn” old patterns of behaviors and practices (Starbuck, 1992), shifting their existing cognitive frames. For example, in the health care sector a learning mindset requires a cultural shift from a focus on performance (Nembhard et al., 2020) and short-term gains that many health care organizations have been driven and incentivized to adapt. It is therefore important that organizations maintain continued investment in fostering positive cultural change (Fiol & Lyles, 1985) (e.g., creating opportunities for cross-functional working and developing clear superordinate goals). To promote constant learning, organizations can incentivize evaluation and reflection, and should continually foster dialogue and feedback loops throughout the organization to spotlight issues before they grow.

### Limitations and Considerations

We utilize a rich dataset to demonstrate the types of adaptations hospitals made in the face of the COVID-19 crisis and the associated capabilities they needed to cope with deep uncertainty. However, because we focused on cataloguing adaptations and offering insights at an aggregate level (i.e., for hospitals more generally) we sacrifice offering a more in-depth analysis at the organizational level. Future research could further tease out the adaptations and lessons learned across and between hospitals to offer meaningful insights into how hospitals with different characteristics (size, academic versus general) adapt and what specific challenges they face. In addition, research that takes a similar approach in different countries and/or cultural contexts would be a welcome addition to our findings. How hospitals in countries with different system characteristics responded would help to identify important boundary conditions and identify additional or different capabilities needed across different systems.

## Conclusion

To remain flexible amid their increasingly uncertain and volatile environments, organizations require dynamic capabilities to sense, seize, and transform. The five categories of adaptations hospitals make in light of the COVID pandemic (i.e., reorganization, decision-making, human resources, material resources, and planning) reveal how these abilities can be developed in resource-constrained environments, such as health care. Organizations can sense by developing a long-term orientation and better integrating their operational core and technostructures and by investing in better forecasting capabilities. They can seize through building collective slack and generating an elastic resource-base and they can transform by adopting a learning mind-set and engaging in unlearning to help foster cultural change. Developing these capabilities can help organizations to be more resilient in facing the uncertainties of the future.

## Supplemental Material

sj-docx-1-mcr-10.1177_10775587211057416 – Supplemental material for To Uncertainty and Beyond: Identifying the Capabilities Needed by Hospitals to Function in Dynamic Environments Click here for additional data file. Supplemental material, sj-docx-1-mcr-10.1177_10775587211057416 for To Uncertainty and Beyond: Identifying the Capabilities Needed by Hospitals to Function in Dynamic Environments by Rachel Gifford, Bram Fleuren, Frank van de Baan, Dirk Ruwaard, Lieze Poesen, Fred Zijlstra and Daan Westra in Medical Care Research and Review
