# Supplementary material for: To Uncertainty and Beyond: Identifying the Capabilities Needed by Hospitals to Function in Dynamic Environments
Source: Med Care Res Rev. 2021 Nov 21;79(4):549–61. doi: 10.1177/10775587211057416 (PMC9218407; doi:10.1177/10775587211057416)
Supplement: sj-docx-1-mcr-10.1177_10775587211057416 – Supplemental material for To Uncertainty and Beyond: Identifying the Capabilities Needed by Hospitals to Function in Dynamic Environments [file sj-docx-1-mcr-10.1177_10775587211057416.docx]

**Supplement 1**

**Research data statement**

**Included Research Data:** Adaptation Catalogue

**Short description:** In our manuscript entitled “To uncertainty and beyond: Identifying the capabilities needed by hospitals to function in dynamic environments” we link to an external opensource database which contains our original research data. Through in-depth analyses of crisis team minutes, internal documentation and in-depth interviews with key stakeholders across the studied region, we compiled an extensive list of adaptations made by hospitals in the wake of the COVID-19 crisis. The hosted data is an adaptation catalogue compiled from our raw data (interviews and archival materials) across all five case organizations. It has been anonymized in line with confidentiality agreements and is made available in both Dutch and English.

**Accessibility**: This adaptation catalogue is freely accessible in line with open data sharing agreements and can be found on Dataverse at <https://doi.org/10.34894/NODI0Q>. For additional transparency, we have included this citation in the manuscript and the raw data is available upon request.
